# How gender and low mental health literacy are related to unmet need for mental healthcare: a cross-sectional population-based study in Sweden

**DOI:** 10.1186/s13690-023-01228-7

**Published:** 2024-01-25

**Authors:** Sara Blom, Frida Lindh, Andreas Lundin, Bo Burström, Gunnel Hensing, Jesper Löve

**Affiliations:** 1 School of Public Health and Community Medicine, Institute of Medicine, University of Gothenburg, Box 453, 405 30 Gothenburg, Sweden; 2 Centre for Epidemiology and Community Medicine, Region Stockholm, Solnavägen 1E, 104 31, Stockholm, Sweden; 3 Department of Global Public Health, Karolinska Institutet, 171 77 Stockholm, Sweden

**Keywords:** Mental health literacy, Health literacy, Unmet need, Barriers to care, Mental health services, Mental disorders, Depression, Anxiety disorders, Gender, Masculinity

## Abstract

**Background:**

Men are more likely to have unmet need for mental healthcare than women. However, an under-investigated aspect of the gender difference is the role of mental health literacy. This study investigated how combinations of gender and mental health literacy were related to two indicators of unmet need: not perceiving a need for mental healthcare despite poor mental health, and refraining from seeking mental healthcare.

**Methods:**

This cross-sectional study was based on a questionnaire sent to a general population sample, aged 16–84 years, in Stockholm County, Sweden, in 2019. Of the 1863 respondents (38%), 1563 were included (≥18 years). The sample was stratified into four groups, men and women with low or high mental health literacy, using the third quartile of the Mental Health Knowledge Schedule. The likelihood of not perceiving a need for mental healthcare and refraining from seeking mental healthcare, at any time in life, were investigated by calculating odds ratios with 95% confidence intervals.

**Results:**

Men with low mental health literacy were most likely to not perceive a need for mental healthcare, also when adjusting for age, education, and poor mental health (OR 5.3, 95% CI 3.6–7.7), and to refrain from seeking mental healthcare, also when adjusting for age and education (OR 3.3, 95% CI 1.7–6.4), followed by men with high mental health literacy (OR 1.9, 95% CI 1.5–2.4, and OR 1.5, 95% CI 1.0-2.2) and women with low mental health literacy (OR 1.9, 95% CI 1.2–2.9, and OR 2.1, 95% CI 1.1–3.9). Women with high mental health literacy were least likely (reference group).

**Conclusion:**

The results show differences in the likelihood of unmet need for mental healthcare based on combinations of gender and mental health literacy level, with men having low mental health literacy being most at risk, and women with high mental health literacy being least at risk. This challenges generalisations of a gender difference in unmet need by showing heterogeneity among men and women based on mental health literacy. Men with low mental health literacy may be particularly in need of targeted interventions to reduce potential individual and societal consequences of their unmet need.

**Supplementary Information:**

The online version contains supplementary material available at 10.1186/s13690-023-01228-7.

**Table:** 

**Text box 1.** Contributions to the literature
• This study adds to the literature by problematizing the common assumption that men are more likely than women to have unmet need for mental healthcare, by showing large within-group differences among men and among women based on mental health literacy. Mental health literacy refers to knowledge that helps in recognising and managing mental disorders.
• Men with low mental health literacy faced the highest risk of not having their mental healthcare needs met.
• Future research should investigate why this is the case, by filling the research gap on how societal expectations of masculinity and level of mental health literacy are connected.

## Background

Depression and anxiety disorders have a lifetime prevalence of 14–15% in high-income countries [1, 2]. Even if the prevalence is two times higher among women than men [1, 2], men are more likely to have unmet need for mental healthcare [3–5]. This is problematic considering the benefits of receiving treatment and the risk for deterioration of symptoms, also among those with milder conditions [6–8].

Unmet need for mental healthcare can occur at several steps on the pathway to healthcare [4, 9]. The clinical need for mental healthcare, i.e. the presence of disabling, persistent, and treatable symptoms [10] corresponding to a mental disorder, has to be transformed into the perception of need for care and the action of seeking care [9]. In high-income countries, between 16 and 51% of those with symptoms corresponding to depression or anxiety disorders do not perceive a need for care [11]. This is a major barrier to mental healthcare [12]. Among those who perceive a need, many refrain from seeking care [13, 14]. Even in Sweden, a country with a universal healthcare system, a study showed that 29% of those who perceived a need for mental healthcare had refrained from seeking it [4]. The study showed that the most common reasons were thinking that the problem would resolve by itself, that the care would not help, or not knowing where to turn for help [4]. Both not perceiving a need, despite symptoms indicating a clinical need for mental healthcare, and refraining from seeking mental healthcare have been found to be more common among men than women in Sweden [4].

Men’s higher likelihood of unmet need for mental healthcare is suggested to be one of the explanations for men’s higher alcohol consumption, possibly a sign of self-medication, and men’s higher risk for premature death and suicide [15, 16]. Men’s higher likelihood of unmet need may be explained by gender norms in the society defining individuals’ possible actions and their consequences [17–19]. Dominant masculinity norms encourage men to be self-reliant, competitive, and decisive, and discourage feminine traits such as being weak, emotional, or shy [18, 20, 21]. This can lead to denial of symptoms and reluctance to seek healthcare, especially for depression and anxiety disorders that are traditionally connected to being feminine [18, 19, 22]. It can also lead to gender bias in the healthcare system, as indicated by signs of under-diagnosis and under-treatment of depression in men [23–25].

An under-investigated aspect of the gender difference in unmet need for mental healthcare is the role of mental health literacy. Mental health literacy has been defined as “knowledge and beliefs about mental disorders that aid their recognition, management and prevention” [26]. The concept includes the ability to recognise symptoms of mental disorders, knowledge about available treatments and their effectiveness, and attitudes that encourage appropriate care-seeking [27]. Previous studies have found that those with lower mental health literacy have a lower intention to seek care, lower actual mental healthcare-seeking, and lower subsequent use of psychotherapy and psychiatric medications [28–30], indicating a higher level of unmet need for mental healthcare in this group.

Men have been found to have lower mental health literacy than women [31]. For example, men have been found to have a lower ability to recognise symptoms of depression in a fictive vignette [32, 33], and to have more stigmatising attitudes towards depression [34, 35]. The lower mental health literacy among men could be explained by gender norms [36], as mental health literacy should be understood not only as an individual skill but also as a reflection of social structures [37–39]. However, there are differences in mental health literacy among men and women with potential implications for the risk of unmet need for mental healthcare. For example, persons with lower education have been found to have lower mental health literacy [31]. A study found that men adhering to traditional masculinity norms had lower communicative and interactive health literacy, although this study did not investigate mental health literacy specifically [40]. However, to our knowledge, no study has investigated how different combinations of gender and mental health literacy are related to unmet need for mental healthcare. Such studies are important as differences among men and women are often as large as differences between men and women [41].

The overall aim of this population-based study in Sweden was to investigate how different combinations of gender and mental health literacy were related to two indicators of unmet need for mental healthcare: not perceiving a need for mental healthcare despite controlling for poor mental health, and refraining from seeking mental healthcare when perceiving a need for it. We assumed that the combined effect of gender and mental health literacy would be of importance for unmet need for mental healthcare, with men with low mental health literacy being most likely not to perceive a need for care and to refrain from seeking care, and women with high mental health literacy being least likely. We expected men with high mental health literacy and women with low mental health literacy to be placed in the middle compared to the above-mentioned groups.

## Methods

### Study population

**Fig. 1:**
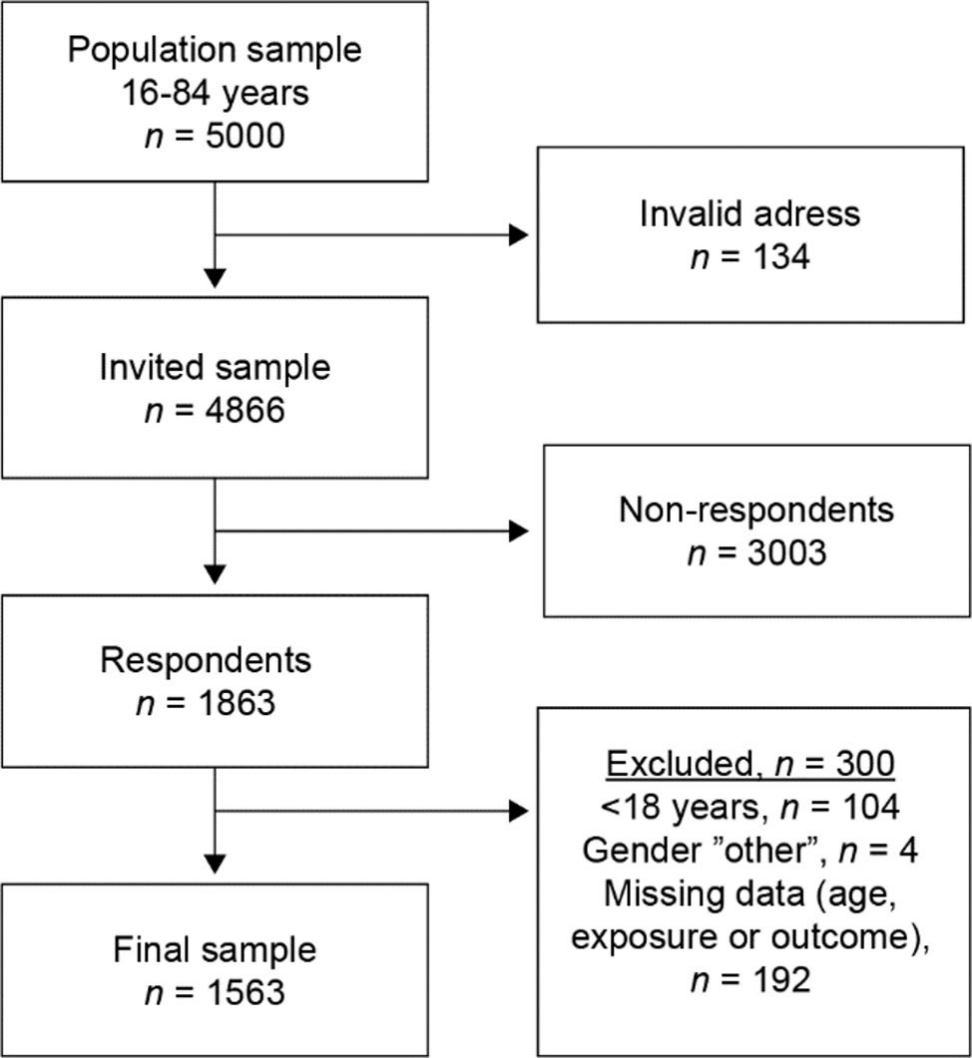
Flow chart of the final sample

We used a questionnaire sent to a general population sample of persons aged 16–84 years in Stockholm County, Sweden, in 2019. Stockholm County had 2.3 million inhabitants in 2018, living either in the capital or in surrounding urban and rural areas [42]. Data was collected by the Centre for Epidemiology and Community Medicine, Region Stockholm. A random sample of 5000 individuals was extracted by the Swedish Tax Agency: 4000 individuals aged 16–84, and an additional 1000 individuals aged 16–29. Those aged 16–29 were oversampled due to expected lower response rates in these groups [43]. An invitation to participate, including the anonymous questionnaire, was sent by mail in May 2019. Two reminders were sent out to everyone (irrespective of whether they had answered or not, information which was unknown due to the anonymous design). Data on names and addresses were deleted after the reminders were sent. Due to invalid addresses, 134 invitations were returned. Of the 4866 individuals who received the invitation, 1863 participated, resulting in a 38% response rate (69% replied by mail, 31% through a web page). Non-participation analyses showed lower participation in men, younger persons, and those born outside Sweden. In this study, those < 18 years were excluded as minors’ mental healthcare-seeking is partly dependent on their caregivers [38]. Those reporting gender as “other” were also excluded as the group was too small to allow statistical analyses. The final sample consisted of 1563 individuals (Fig. 1).

### Variables

#### Exposures

For this study, the variables of gender and mental health literacy were combined into four exposure groups: (1) men with low mental health literacy, (2) men with high mental health literacy, (3) women with low mental health literacy and (4) women with high mental health literacy.

Gender (woman, man) was self-reported. Mental health literacy was measured using the first section of the Mental Health Knowledge Schedule (MAKS), covering the respondents’ perceptions about care-seeking, the effectiveness of treatment, recovery, and employment for people with mental health problems (e.g. “ Medication can be an effective treatment for people with mental health problems”) [44]. The six items were followed by a six-point Likert scale with response alternatives ranging from “strongly agree” (1 point) to “strongly disagree” (5 points). The alternative “Don’t know” was coded as neutral [3]. The total score ranged from 6 to 30 with a higher score indicating lower mental health literacy. Item 6 (“Most people with mental health problems go to a health care professional to get help”) was reverse coded to reflect that “strongly disagree” was the most correct response. MAKS has shown fair one-week test-retest reliability, poor to moderate internal consistency, and excellent content validity [44, 45]. For this study, only those that had valid data on all items on MAKS were included. In the absence of an established definition of low mental health literacy, the total score of MAKS was dichotomised into low versus high mental health literacy using the third quartile in the total sample as a threshold. The Swedish translated version was used [46].

#### Outcomes

Unmet need for mental healthcare at any time in life was operationalised using two indicators: (1) not perceiving a need for mental healthcare despite adjusting for current poor mental health, or (2) refraining from seeking mental healthcare when perceiving a need for it. These indicators were measured by the question “Have you at any time felt so mentally unwell that you felt a need to seek care?” with the response alternatives “Yes, and I have finished treatment”, “Yes, and I have ongoing treatment”, “Yes, but I did not seek care” and “No”. Not perceiving a need for care was defined as answering “No” versus “Yes” (“Yes, and I have finished treatment”, “Yes, and I have ongoing treatment”, or “Yes, but I did not seek care”). Refraining from seeking mental healthcare was defined as answering “Yes, but I did not seek care” versus “Yes, and I have finished treatment” or “Yes, and I have ongoing treatment”.

#### Covariates

Factors that were empirically or theoretically connected to gender, mental health literacy, and the respective outcome were chosen as covariates.

For analyses on not perceiving a need for mental healthcare current mental health was included as a covariate as good mental health is associated with not perceiving a need for mental healthcare [3]. Current mental health was measured using the 12-item Swedish version of the General Health Questionnaire (GHQ-12). GHQ-12 was developed to detect common non-psychotic psychiatric disorders [47, 48]. Items were coded according to the Standard GHQ score (0-0-1-1), with higher scores indicating poorer mental health on a scale from 0 to 12. Those that had missing data on any item were excluded from the analyses (n = 24). A cut-off of ≥ 3 was used to indicate current poor mental health. This cut-off has previously indicated psychological distress [49–51], and corresponded to depression, anxiety, or adjustment disorder [52], all of which imply a clinical need for mental healthcare.

In addition, age and level of education were included as covariates for both outcomes. Lower age has been found to be associated with better mental health literacy [31, 53], and a higher likelihood of not perceiving a need for mental healthcare, and of refraining from seeking it [3, 54]. Lower education has been found to be associated with lower mental health literacy [31, 55], male gender [56], and a higher likelihood of not perceiving a need for mental healthcare, and of refraining from seeking it [3, 4]. Age and education were self-reported. Age was categorised into 19–30, 31–50, 51–64, 65–84 years. Level of completed education was categorised into primary or less, secondary, and university education.

### Statistical analyses

To compensate for the oversampling of those aged 18–29 and gender and age differences in study participation, the analyses were conducted on weighted data. The sample was weighted based on the gender and age distribution in the general population in Stockholm County, in 2018. The weight variable was calculated by dividing the proportion in the general population with the corresponding proportion in the sample [57]. For example, the proportion of men aged 29 years in the general population was divided by the proportion of men aged 29 years in the study sample, giving this group a weight value of 1.57 (0.011/ 0.007 = 1.57). Sample characteristics were presented as both weighted and unweighted frequencies (n=) and proportions (%). All other results were presented based on weighted data only. The third quartile on MAKS was calculated to establish the cut-off for low mental health literacy. Gender differences in mental health literacy and the two outcomes (not perceiving a need for mental healthcare, and refraining from seeking it) were investigated using proportional differences (%) with 95% confidence intervals (95% CI) [58]. The sample was then stratified into groups: (1) men with low mental health literacy, (2) men with high mental health literacy, (3) women with low mental health literacy, and (4) women with high mental health literacy (reference group). Group differences in the likelihood of having the outcomes were investigated by using frequencies and proportions. Bivariate and multivariable logistic regression analyses were then conducted, presented as crude and adjusted odds ratios (OR) with 95% CIs. Lastly, sensitivity analyses were conducted to (1) investigate if the main results were robust for changes in the definition of low mental health literacy, (2) challenge the use of MAKS by including only some of the items, (3) investigate if the main results persisted using the unweighted data. Analyses were conducted using IBM SPSS Statistics, version 27.

## Results

### Characteristics of the sample

The characteristics of the total sample, and the sample stratified by gender, are shown in Table 1. In the total sample (n = 1563), a higher proportion of women had current poor mental health, compared to men.

### Not perceiving a need for care, refraining from seeking care, and low mental health literacy

**Table 1:** Characteristics of the sample, stratified by gender. Frequencies (n) and proportions (%)

	Total (Unweighted n = 1563) Weighted n = 1563		Men (Unweighted n = 659) Weighted n = 782		Women (Unweighted n = 904) Weighted n = 781	
	(n) n^ab^	(%) %^ac^	n^ab^	%^ac^	n^ab^	%^ac^
**Age, years**						
18–30	(405) 352	(26) 23	179	23	173	22
31–50	(438) 594	(28) 38	303	39	291	37
51–64	(319) 330	(20) 21	166	21	164	21
65–84	(401) 286	(26) 18	134	17	152	20
**Education**						
Primary or less	(169) 139	(11) 9	66	8	73	9
Secondary	(512) 496	(33) 32	266	34	231	30
University	(878) 922	(56) 59	447	57	475	61
**Current mental health**						
Poor	(392) 391	(25) 25	166	22	224	29

^a^ Weighted data based on the gender and age distribution in Stockholm County. ^b^ As the weighted frequencies were rounded to the nearest integer, frequencies in sub-groups do not always sum up to the total frequency. ^c^ Column proportions. Valid proportions, missing values excluded.

In the total sample, 53% had not perceived a need for mental healthcare at any time in life. Among those who had perceived a need for mental healthcare, 26% had refrained from seeking it. Not perceiving a need for mental healthcare, and refraining from seeking mental healthcare was more common among men than women (62% versus 45%, and 31% versus 22%, Table 2). Among the sample, 22% were defined to have low mental health literacy (≥16 on MAKS based on the third quartile). Low mental health literacy was more common among men than women (27% versus 18%, Table 2).

### Combinations of gender and mental health literacy

**Table 2:** Not perceiving a need for mental healthcare, refraining from seeking mental healthcare, and having low mental health literacy, by gender. Weighted frequencies (n), proportions (%), and proportional differences (%, 95% CI) between men and women^a^

	Total n = 1563		Men n = 782		Women n = 781		Men versus women Differences between proportions
	n	%^d^	n	%^d^	n	%^d^	% (95% CI) ^e^
Had not perceived a need for care, at any time^b^	834	53	486	62	348	45	18 (13–22)
Perceived a need for care^b^	729	47	296	38	433	55	
Refrained from seeking care, at any time^c^	189	26	93	31	96	22	9 (3–16)
Sought care^c^	540	74	203	69	337	78	
Low mental health literacy	346	22	209	27	137	18	9 (5–13)
High mental health literacy	1217	78	573	73	644	82	

^a^ Weighted data based on the gender and age distribution in Region Stockholm, Sweden. ^b^ Among the total sample. ^c^ Among those who had perceived a need for care at any time in life. ^d^ Column proportions. ^e^ The difference between the proportion among men compared to the proportion among women, and the 95% confidence interval of the difference. Rounded to the nearest integer.

Stratifying the sample based on combinations of gender and mental health literacy showed that not perceiving a need for mental healthcare was most common among men with low mental health literacy (76%), followed by women with low mental health literacy (59%), men with high mental health literacy (57%), and women with high mental health literacy (42%, Table 3). This pattern was also observed when including only those with current poor mental health (see Additional file 1). Refraining from seeking mental healthcare was most common among men with low mental health literacy (46%), followed by women with low mental health literacy (37%), men with high mental health literacy (28%), and women with high mental health literacy (20%, Table 3).

### Logistic regression analyses

**Table 3:** Not perceiving a need for mental healthcare, or refraining from seeking it, at any time in life, by gender combined with mental health literacy. Weighted frequencies (n) and proportions (%)^a^

	Total	Not perceived a need for care^b^ n = 834		Refrained from seeking care^c^ n = 189	
	n	n	%^d^	n	% ^d^
Men, low mental health literacy	209	159	76	23	46
Men, high mental health literacy	573	327	57	70	28
Women, low mental health literacy	137	81	59	21	37
Women, high mental health literacy	644	267	42	75	20

^a^ Weighted data based on the gender and age distribution in Stockholm County, Sweden. ^b^ Among the total sample.^c^ Among those who had perceived a need for care at any time in life. ^d^ Row proportions.

Logistic regression analyses showed that compared to women with high mental health literacy (reference), all other groups were more likely to not perceive a need for mental healthcare. This was true even when adjusting for age, education, and current mental health: Men with low mental health literacy were most likely to not perceive a need for care (OR 5.3, 95% CI 3.6–7.7), followed by men with high mental health literacy (OR 1.9, 95% CI 1.5–2.4), and women with low mental health literacy (OR 1.9, 95% CI 1.2–2.4, Table 4).

Compared to women with high mental health literacy, all other groups were also more likely to refrain from seeking mental healthcare, in both crude and adjusted analyses. Men with low mental health literacy were the most likely to refrain from seeking care (OR 3.3, 95% CI 1.7–6.4), followed by women with low mental health literacy (OR 2.1, 95% CI 1.1–3.9), and men with high mental health literacy (OR 1.5, 95% CI 1.0-2.2), adjusting for age and education (Table 4).

### Sensitivity analyses

**Table 4:** Likelihood of not perceiving a need for mental healthcare, or refraining from seeking it, at any time in life. Multivariable logistic regression analyses. Crude and adjusted odds ratios (OR) with 95% confidence intervals (95% CI)^a^

	Not perceived a need for care^b^			Refrained from seeking care^c^	
	OR (95% CI)			OR (95% CI)	
	Crude	Adjusted^d^		Crude	Adjusted^e^
Men, low mental health literacy	4.5 (3.2–6.5)	5.3 (3.6–7.7)		3.4 (1.9–6.3)	3.3 (1.7–6.4)
Men, high mental health literacy	1.9 (1.5–2.3)	1.9 (1.5–2.4)		1.6 (1.1–2.3)	1.5 (1.0–2.2)
Women, low mental health literacy	2.0 (1.4–2.9)	1.9 (1.2–2.9)		2.4 (1.3–4.3)	2.1 (1.1–3.9)
Women, high mental health literacy	1	1		1	1

^a^ Weighted data based on the gender and age distribution in Stockholm County, Sweden. ^b^ Among the total sample. ^c^ Among those who had perceived a need for care at any time in life. ^d^ Adjusted for age, education, and current mental health. ^e^ Adjusted for age and education.

Three sensitivity analyses were carried out: Firstly, even when using the MAKS median (≥14) as the cut-off for low mental health literacy the results were in line with those reported above (see Additional file 2). Secondly, we challenged the MAKS by excluding item 6 as the item was potentially problematic due to low item-to-total correlation (“Most people with mental health problems go to a health care professional to get help”). However, the results were in line with those reported above (see Additional file 3). Thirdly, we investigated whether the main results persisted also when using the unweighted data, and these results were also in line with those reported above (results not shown).

## Discussion

This population-based study in Sweden showed differences in the likelihood of unmet need for mental healthcare based on combinations of gender and mental health literacy. The results are interesting considering that Sweden is a relatively gender-equal country with a universal healthcare system striving for equal access to healthcare for all [59, 60]. Men with low mental health literacy had the highest likelihood of unmet need, followed by men with high mental health literacy and women with low mental health literacy. Women with high mental health literacy had the lowest likelihood. This was true for both indicators: not perceiving a need for mental healthcare despite controlling for current poor mental health, which is previously found to correspond to depression, anxiety, or adjustment disorder [52], and refraining from seeking mental healthcare when perceiving a need for it. The results persisted even when controlling for education level, which is previously known to correspond to mental health literacy [31, 32, 55]. The results challenge simplistic generalisations of gender differences in unmet need by showing heterogeneity among men and women based on mental health literacy [19, 41].

### Men with low mental health literacy

Compared to all other groups, men with low mental health literacy had the highest likelihood of unmet need for mental healthcare. The results are in line with previous research that has shown that both male gender and low mental health literacy are factors associated with unmet need [3–5, 28–30]. However, to our knowledge, no previous study has investigated their combined effect. The high likelihood of unmet need among men with low mental health literacy could potentially be explained by combined exposure to masculinity norms and low mental health literacy. These factors could together and separately discourage mental healthcare-seeking: Firstly, traditional masculinity norms of e.g., strength and independence, have been found to be associated with denial of symptoms of depression and refraining from seeking care [18, 19]. Secondly, persons with low mental health literacy are less likely to be able to recognise symptoms of a mental disorder, and to know about available and effective treatment [27]. Thirdly, men with low general health literacy have been found to be more likely to adhere to traditional masculinity norms [61], but research focusing on mental health literacy is lacking. The impact of masculinity norms on general health literacy is under-researched globally [36], and research on mental health literacy is especially scarce. Therefore, to better understand the found combined effect of male gender and low mental health literacy on unmet need, research is needed to explore how masculinity norms and mental health literacy are intertwined, especially in relatively gender-equal countries such as Sweden.

### Similarities and differences between men and women

Men with high mental health literacy and women with low mental health literacy had a similar likelihood of unmet need for mental healthcare. Both groups had an almost twofold higher likelihood of not perceiving a need for care, and of refraining from seeking it, compared to women with high mental health literacy (Table 4). This highlights the importance of investigating not only differences between men and women but also similarities between groups of men and women [41] to broaden the understanding of unmet need.

The higher likelihood of unmet need among men with high mental health literacy compared to women with high mental health literacy confirms the previously found gender differences in unmet need [3–5]. However, it is interesting that the gender difference persisted even with the assumed protection of high mental health literacy among men, indicating that other factors are involved. The gender difference was most pronounced regarding not perceiving a need for mental healthcare, but less pronounced regarding refraining from seeking care: the adjusted results was not statistically significant, although it leans towards a difference (OR 1.5, 95% CI 1.0–2.2), Table 4). It is likely that adherence to some aspects of masculinity norms, such as self-reliance, hinder even privileged men as those with high mental health literacy to perceive themselves as being in need of care. For example, masculinity norms can hinder men from identifying as being depressed or anxious to avoid perceiving themselves as feminine [18]. However, those who have already perceived themselves as being in need of care may have overcome such gendered barriers, which could explain the less pronounced gender difference in refraining from seeking mental healthcare when having perceived a need for it. Overall, the results from this study highlight that increasing men’s mental health literacy will not be enough to reduce men’s unmet need for mental healthcare. Probably, interventions focusing on both mental health literacy and masculinity norms are needed.

### The reference group: women with high mental health literacy

Women with high mental health literacy were used as the reference group. Their relatively low level of unmet need could potentially be seen as the ideal, as the health status of those that are better off can be used as the goal for groups that are worst off in public health interventions [62]. Still, 22% of the women with high mental health literacy and current poor mental health had not perceived a need for mental healthcare (see Additional file 1), and 20% of those who had perceived a need had refrained from seeking care (Table 3). This indicates a notable unmet need even among these women, but could also be a sign of an overestimation of unmet need for mental healthcare in this study. Some of those that did not perceive a need for care, or refrained from seeking it, may have had transient distress that did not require treatment [63, 64].

### Mental health literacy as a reflection of social structures

Mental health literacy could be understood as a reflection of social structures and not only as an individual skill or trait [37–39]. Mental health literacy refers to knowledge and beliefs that helps in recognising and managing mental disorders [27], but obviously individuals’ knowledge and beliefs are affected by the social structures they live in e.g., gender norms, the design of the health care system, and social inequalities based on for example education and class. To better understand the results from the current study, more knowledge is needed on how social structures interact with individuals’ mental health literacy and enable or hinder them from perceiving a need for care, and subsequently seeking it. Previous research found that health literacy works as mediator for the relationship between low socioeconomic position and poor health outcomes, i.e., reinforce health inequalities [65]. For example, lower education is associated with both lower mental health literacy [31, 55] and a higher risk for unmet need for mental healthcare [3, 4]. However, based on our findings, mental health literacy also seems to work independently from education level (Table 4). The results suggest that low mental health literacy adds disadvantage in regard to unmet need, regardless of education level, especially for men. Low mental health literacy may also reflect inequalities produced and reproduced by social institutions such as the healthcare system. For example, the primary healthcare system in Sweden has been criticised for favouring affluent groups with a high demand for care [66]. Men’s lower mental health literacy could also mirror the gendered nature of the healthcare system. For example, two qualitative studies from Sweden recently found that healthcare personnel working with men’s sexual health often held traditional and stereotypical notions of masculinity, potentially reflected in their caregiving [67], and that the sexual healthcare in itself was constructed as a feminine and designed for women, which could discourage men from seeking care [68]. Research is needed to investigate if such gender bias exists also within the framing and organisation of the mental healthcare in Sweden, and how it may affect men’s mental health literacy, e.g., by reinforcing scepticism to the mental healthcare and its treatments. In sum, the relations between mental health literacy, unmet need, and social structures as gender norms, are complex. The results from this study suggests that mental health literacy is a gendered phenomenon that impacts individuals’ pathways to receiving the mental healthcare that they need [69].

### Methodological considerations

A strength of this study is the use of two indicators of unmet need for mental healthcare. Not perceiving a need for mental healthcare, despite controlling for current poor mental health corresponding to depression, anxiety, or adjustment disorder [52], is based on the definition of need as fulfilling clinical criteria for a diagnosis [70]. Refraining from seeking mental healthcare, despite perceiving a need for it, is based on the definition of need as individuals’ perceptions. Previous studies have usually focused on individuals’ perception of need only [71], overlooking the fact that low perceived need for mental healthcare is a major barrier to mental healthcare [3].

Another strength of this study is the use of an established cut-off on GHQ-12 (≥ 3) to indicate a current poor mental health [52], implying a clinical need for mental healthcare. However, the GHQ-12 questionnaire cannot fully replace the assessment of healthcare need by a clinician, as they also assess how severe, persistent, and treatable symptoms are [70]. This has implications for the study’s results. For example, women with high mental health literacy could have had a higher clinical need for care compared to the other groups, which could explain their higher perceived need and mental healthcare-seeking [5, 12].

A limitation is that the measure of *perceived need* referred to “any time in life” but the indicator implying *clinical need*, i.e. GHQ-12, referred to “the previous weeks”. However, ideally, clinical need in the previous weeks should translate into reporting a lifetime perceived need for mental healthcare [59]. Therefore, the different time periods should not have affected the results, but future studies should measure clinical and perceived need within the same time periods to better capture their discrepancy.

Another potential limitation is that the measure of mental health literacy (MAKS) only covers some of the attributes included in Jorm’s definition [26]. Based on our interpretation, items 1–6 mainly cover the part relating to “knowledge and beliefs about the professional help available” [27] whereas other aspects, such as knowledge about self-help, are not covered. Also, in the absence of an established definition of mental health literacy [72], we dichotomised mental health literacy using the third quartile based on the sample’s distribution of MAKS scores. However, this cut-off is experimental and context-bound and cannot be generalised to other contexts. Therefore, the study’s result should be challenged in future studies using other measures and definitions of mental health literacy.

Another limitation is the participation rate of 38% since it resulted in low statistical power for subgroup analyses and potentially introduced bias due to differences between participants regarding the associations between exposures and outcomes [73]. Still, the response rate is in line with the declining participating rate in epidemiological studies and previous research has found little evidence for extensive bias based on non-participation [73]. If anything, we might have underestimated the prevalence of unmet need for mental healthcare among men, as a previous study found that male non-participants had lower healthcare utilisation than male participants [74].

Finally, it should be highlighted that we are limited by the cross-sectional and observational design of the study, and conclusions about causality should be drawn with caution.

### Implications

The found differences suggest that interventions aiming to reduce unmet need for mental healthcare might be too crude if a sole focus is on men’s higher likelihood of unmet need. Differences in mental health literacy among men and women should be considered. An urgent task is to target men with low mental health literacy, even though women with low mental health literacy and men with high mental health literacy should not be ignored. Still, to provide more accurate interventions, more research is needed. Firstly, we need to disentangle the characteristics of these groups, e.g., who are the men with low mental health literacy, and how can they be reached? Secondly, future research needs to explore the relations between masculinity norms and low mental health literacy in Sweden. Thirdly, longitudinal studies are needed to investigate whether potential consequences of having an unmet need for mental healthcare, e.g., high alcohol consumption, and suicidality, differ between the groups.

## Conclusions

This population-based study in Sweden showed differences in likelihood of unmet need for mental healthcare based on combinations of gender and mental health literacy. Men with low mental health literacy had the highest likelihood of unmet need. They were followed by men with high mental health literacy and women with low mental health literacy, groups which had a similar likelihood. Women with high mental health literacy had the lowest likelihood. This was true for both indicators of unmet need: not perceiving a need for mental healthcare despite adjusting for poor mental health, and refraining from seeking mental healthcare despite perceiving a need for it. The results challenge simplistic generalisations of gender differences in unmet need by showing heterogeneity among men and women based on mental health literacy. The groups with a higher likelihood, especially men with low mental health literacy, may need targeted interventions to reduce the individual and societal consequences of their unmet need for mental healthcare.

### Electronic supplementary material

Below is the link to the electronic supplementary material.

Supplementary Material 1. File name: Additional file 1.pdf. Title of data: Additional file 1. Supplementary table. Description of data: Sensitivity analyses among those that had current poor mental health.

Supplementary Material 2. File name: Additional file 2.pdf. Title of data: Additional file 2. Supplementary table. Description of data: Sensitivity analyses using the median as the cut-off for low mental health literacy.

Supplementary Material 3. File name: Additional file 3.pdf. Title of data: Additional file 3. Supplementary table. Description of data: Sensitivity analyses using MAKS item 1–5, excluding item 6.

## Data Availability

The datasets used and analysed during the current study are available from the corresponding author on reasonable request.

## List of abbreviations

MAKS: Mental Health Knowledge Schedule
GHQ-12: General Health Questionnaire
95% CI: 95% confidence intervals
OR: Odds ratios
