# Supplementary material for: How gender and low mental health literacy are related to unmet need for mental healthcare: a cross-sectional population-based study in Sweden
Source: Arch Public Health. 2024 Jan 25;82:12. doi: 10.1186/s13690-023-01228-7 (PMC10809616; doi:10.1186/s13690-023-01228-7)
Supplement: Supplementary file 1 — Supplementary Material 1. File name: Additional file 1.pdf. Title of data: Additional file 1. Supplementary table. Description of data: Sensitivity analyses among those that had current poor mental health. [file 13690_2023_1228_MOESM1_ESM.docx]

| Additional file 1. Supplementary table. Sensitivity analyses among those that had current poor mental health. Not perceiving a need for mental healthcare, at any time in life, by gender combined with mental health literacy. Weighted frequencies (n) and proportions (%)^a^. | | | |
| --- | --- | --- | --- |
|  | Total | Had not perceived a need for care  n=120 | |
| Current poor mental health^b^ | n | n | %^c^ |
| Men, low mental health literacy | 46 | 30 | 65 |
| Men, high mental health literacy | 120 | 35 | 29 |
| Women, low mental health literacy | 36 | 13 | 36 |
| Women, high mental health literacy | 188 | 42 | 22 |

^a^ Weighted data based on the gender and age distribution in Stockholm County, Sweden

^b^ Defined using cut-off ≥3 on the General Health Questionnaire (GHQ-12)

^c^ Row proportions.
