# Supplementary material for: How gender and low mental health literacy are related to unmet need for mental healthcare: a cross-sectional population-based study in Sweden
Source: Arch Public Health. 2024 Jan 25;82:12. doi: 10.1186/s13690-023-01228-7 (PMC10809616; doi:10.1186/s13690-023-01228-7)
Supplement: Supplementary file 2 — Supplementary Material 2. File name: Additional file 2.pdf. Title of data: Additional file 2. Supplementary table. Description of data: Sensitivity analyses using the median as the cut-off for low mental health literacy. [file 13690_2023_1228_MOESM2_ESM.docx]

| Additional file 2. Supplementary table. Sensitivity analyses using the median as the cut-off for low mental health literacy (≥14). Likelihood of not perceiving a need for mental healthcare, or refraining from seeking it, at any time in life. Multivariable logistic regression analyses. Crude and adjusted odds ratios (OR) with 95% confidence intervals (95% CI)^a^. | | | | | |
| --- | --- | --- | --- | --- | --- |
|  | Not perceived a need for care^b^ | |  | Refrained from seeking care^c^ | |
|  | OR (95% CI) | |  | OR (95% CI) | |
|  | Crude | Adjusted^d^ |  | Crude | Adjusted^e^ |
| Men, low mental health literacy | 3.5 (2.6–4.6) | 3.8 (2.8–5.1) |  | 2.9 (1.8–4.6) | 2.8 (1.7–4.5) |
| Men, high mental health literacy | 2.1 (1.6–2.7) | 2.1 (1.6–2.8) |  | 1.4 (0.9–2.2) | 1.3 (0.8–2.2) |
| Women, low mental health literacy | 2.0 (1.5–2.7) | 2.0 (1.4–2.7) |  | 1.8 (1.2–3.0) | 1.7 (1.1–2.9) |
| Women, high mental health literacy | 1 | 1 |  | 1 | 1 |

^a^ Weighted data based on the gender and age distribution in Stockholm County, Sweden.

^b^ Among the total sample.

^c^ Among those who had perceived a need for care at any time in life.

^d^ Adjusted for age, education, and current mental health.

^e^ Adjusted for age and education.
